# Longitudinal Serial Mediation Relationship of Intolerance of Uncertainty, Irrational Happiness Beliefs and Mistake Rumination with Family Communication

**DOI:** 10.1007/s11126-025-10157-3

**Published:** 2025-05-05

**Authors:** Ali Berke Körün, Seydi Ahmet Satıcı

**Affiliations:** Faculty of Education, Department of Psychological Counseling, Yıldız Technical University, Istanbul, Türkiye

**Keywords:** Intolerance of uncertainty, Irrational happiness beliefs, Mistake rumination, Family communication, Longitudinal

## Abstract

The family systems approach suggests that individuals' psychological processes are shaped within the framework of family interactions. Accordingly, the current study examines the long-term effects of intolerance of uncertainty, irrational happiness beliefs and mistake rumination on family communication within the scope of a semi-longitudinal model. The study analyzed the predictive power of intolerance of uncertainty on irrational happiness beliefs and mistake rumination and whether these variables play a mediating role in family communication. The study, which was conducted in two phases at five-month intervals, included 325 adult participants aged 18–51. Longitudinal serial mediation analysis revealed that intolerance of uncertainty increases irrational happiness beliefs and mistake rumination over time, and these processes negatively affect family communication. The findings suggest that individuals' low tolerance for uncertainty may shape long-term psychological and relational dynamics through family interactions. The study emphasizes the importance of holistic interventions to support the functionality of family systems. In particular, it is suggested that short-term systemic family therapy and emotion-focused individual interventions may be effective in strengthening family communication and increasing the psychological resilience of individuals.

## Introduction

During human history, individuals have faced various challenges as part of the struggle for survival. In this struggle process, sometimes human communities and sometimes the natural environment have been significantly affected. Events such as natural disasters, occupational accidents, wars, mass migrations and terrorist attacks in the historical process have caused individuals to experience both tangible and psychological losses. Such events leave deep impacts not only on the individual level but also on social structures and especially on family systems [21]. As the most basic relational structure that shapes the emotional and psychological well-being of individuals, the family can be directly affected by such crisis situations and plays a determining role in the quality of life of its members [43]. Interactions within the family enable individuals to acquire important skills such as rational decision-making processes, emotional regulation skills, and coping skills with stressful situations [9]. The approach that examines the effects of family interactions on individuals'psychological skills is the family systems approach. This approach defines the family as a system that is in constant communication, whose members are in constant interaction and has common interaction areas [23]. The family is a dynamic structure determined by its own rules, boundaries and communication patterns. Rather than focusing on individuals in isolation, the family systems approach emphasizes the family as a unit where each member contributes to and is impacted by the family’s overall dynamics.

According to the family systems approach, there are numerous factors that determine family functioning. These factors interact with each other and include elements such as family support, family resilience and family belonging [36]. One of the main components of family functioning is family communication, which, together with other functions within the family, stands out as an important mechanism that shapes individuals'perceptions of their families. Family communication also contributes to the development of individuals'skills to cope with psychological difficulties [19]. Longitudinal studies reveal that strong family communication increases individuals'general well-being and life satisfaction [31]. Moreover, family communication plays a critical role in times of crisis. For example, a study conducted by [46] during the pandemic showed that family communication has strong relationships with family cohesion, social support and moral support. Similarly, another study found that family communication was a factor supporting family resilience [7]. Buehler et al. [8] demonstrate that increased family communication strengthens interdependence and psychological well-being and reduces feelings of loneliness. In general, family communication appears to have a strong interaction with both other family functions and factors that support individuals'psychosocial well-being.

Family communication quality is an important factor shaping individuals'stress coping strategies and emotional adjustment. However, although family communication is a critical process, it can be affected by various factors. In particular, discomfort in the face of uncertainty can negatively affect communication dynamics within the family [6]. Accordingly, intolerance of uncertainty stands out as an important psychological variable that determines family relationships and overall cohesion. Research shows that intolerance of uncertainty increases health-related anxiety within the family [1]. However, it affects not only family processes but also the emotional mechanisms of individuals. Meta-analysis findings reveal that intolerance of uncertainty has a positive relationship with maladaptive emotions and a negative relationship with adaptive emotions [37]. Deniz [16] found that intolerance of uncertainty played a serial mediating role between self-compassion, fear of COVID-19 and well-being. Similarly, another study shows that intolerance of uncertainty functions as a mediating variable in the relationship between emotional reactivity and well-being [5].

Intolerance of uncertainty may lead individuals to experience higher levels of anxiety and stress in the face of unpredictable situations [45]. This may also affect how individuals evaluate their past experiences and increase their tendency to dwell on mistakes and constantly review them [39]. As a result, mistake rumination may occur in individuals. Research shows that with increased mistake rumination, depression levels increase and overall mental health is negatively affected [14]. Rumination not only affects individual mental health but also contributes to heightened anxiety and depression. These outcomes, in turn, may give rise to health-related problems within the family context [35]. Particularly in the context of mistakes made in daily life, rumination becomes more evident. Individuals may make various mistakes in their daily lives,however, repeatedly thinking about these mistakes triggers the process of mistake rumination. Continuously focusing on past mistakes may cause the individual to develop ruminative thoughts about both himself/herself and his/her cultural context [13]. Findings show that increased mistake rumination increases perfectionism efforts, worry about making mistakes, and anxiety levels [3]. Moreover, mistake rumination may limit individuals'cognitive flexibility by causing them to constantly evaluate their past mistakes. These patterns may shape how individuals define and pursue happiness, often leading to the development of rigid criteria for what constitutes a fulfilling life [42].

Happiness can be defined as the general satisfaction with one's life and the predominance of positive emotions. However, individuals'perception of happiness may differ depending on the beliefs they hold. Irrational happiness beliefs include unrealistic thoughts such as that happiness is only possible under certain conditions, that a constant and perfect state of happiness is sustainable, or that unhappiness is a state that should be completely avoided [49]. Such beliefs can negatively affect emotional adjustment by making it difficult for the individual to cope with inevitable life challenges. Research shows that irrational happiness beliefs reduce individuals'quality of life and have a negative impact on their well-being [22]. Longitudinal studies reveal that such beliefs can affect individuals'well-being levels with momentary fluctuations over time [48]. It is also suggested that irrational happiness beliefs make happiness more fragile and may negatively affect physical health [28]. However, Joshanloo [24] argues that beliefs about happiness are associated with negative personality traits and may play a mediating role for these personality traits. In other words, happiness beliefs not only negatively affect individual well-being, but may also reveal personality traits that reduce individual functioning.

## The Current Study

The modern world is going through a period in which individuals face uncertainty more than ever before. Global pandemics, economic crises, disasters, climate change, political uncertainties and technological transformations constantly reshape people's daily lives and perceptions of the future [2, 15]. In particular, rapidly changing social dynamics make it difficult for individuals to feel secure and may trigger psychological tendencies such as intolerance of uncertainty. Intolerance of uncertainty causes individuals to perceive unpredictable situations as threats and to feel anxiety in the face of uncontrollable circumstances. Beyond individual psychology, this situation can also affect interpersonal relationships and especially communication processes within the family. The family constitutes the first relational framework that the individual establishes with the world and can function as a kind of “safe harbor” in the face of changing social conditions. However, as individuals'intolerance of uncertainty increases, communication dynamics within the family may also be affected. The family systems approach considers the family not only as a structure consisting of individuals, but also as a system that is in constant interaction between its members and has its own internal rules and boundaries [33]. In this context, an individual's cognitive processes and psychological tendencies are shaped not only in his/her inner world but also through relationships within the family. This study aims to examine the longitudinal effect of intolerance of uncertainty, mistake rumination, and irrational happiness beliefs on family communication. Family communication is one of the main mechanisms that directly affect individuals'coping strategies with uncertainty and emotional regulation processes. Therefore, understanding the transformation of the relationship between variables over time may provide important implications for both theoretical perspectives and psychological interventions. Within this context, the following hypotheses (H) will be tested in this study:

- **H1**. Intolerance of uncertainty will longitudinally predict irrational happiness beliefs.
- **H2.** Intolerance of uncertainty will longitudinally predict mistake rumination.
- **H3.** Irrational happiness beliefs and mistake rumination will have a longitudinal serial mediation effect on the relationship between intolerance of uncertainty and family communication.

## Method

### Participants and Procedure

A half-longitudinal study was conducted with five months between each measurement, starting with the first measurement in the second week of September 2024 and ending with the second measurement in the fourth week of February 2025. Ethics committee approval has been obtained from the Yıldız Technical University Institute of Social Sciences. Then, the research data were collected using a web-based form. The link to the online form was sent to the participants to whom the authors had access. All participants provided informed consent before taking part in the study. Participation was voluntary, and no compensation was provided. In the first wave, a total of 379 participants were included. In the second wave, 351 participants completed the research form. Participants from both waves were matched based on their responses, resulting in a final sample of 325 Turkish adults (237 women, 72.9%; 88 men, 27.1%). Participants'ages ranged from 18 to 51 years (M_age_ = 25.11, SD = 3.52). The majority of the participants were single (n = 239, 73.5%).

### Measures

#### Intolerance of Uncertainty Scale

This scale originally developed by Carleton et al. [11] and adapted to Turkish by Sarıçam et al. [38]. This two sub dimensional (prospective anxiety and inhibitory anxiety) scale has twelve items. The total score on a 5-point Likert scale with 1 meaning “*not suitable for me at all*” and 7 being “*totally suitable for me*” can range from 7 to 35. Higher scores on the two factors indicate a high intolerance of uncertainty. In the adaptation study of the scale, the internal consistency coefficient was evaluated as 0.88.

#### Irrational Happiness Beliefs Scale

This scale developed by Yıldırım & Maltby [49] to measure happiness as an absolute by using specific words of “should,” “must,” and “ought to. This one-dimensional scale has three items. The total score on a 7-point Likert scale with 1 meaning “*strongly disagree*” and 7 being “*strongly agree*” can range from 3 to 21. In the validation study of the scale, the internal consistency coefficient was evaluated as 0.84.

#### Mistake Rumination Scale

This scale originally developed by [18] and adapted to Turkish by [25]. This one-dimensional scale has seven items. The total score on a 4-point Likert scale with 1 meaning “*not at all*” and 4 being “*very much*” can range from 7 to 28. Higher scores indicate that participants ruminate more on their mistakes. In the adaptation study of the scale, the internal consistency coefficient was evaluated as 0.82.

#### Family Communication Scale

This scale developed by Geçer & Yıldırım [19] to measure family communication. This one-dimensional scale has six items.. The total score on a 4-point Likert scale with 1 meaning “strongly disagree” and 4 being “strongly agree” can range from 6 to 24. High scores indicate that participants’ families communicate effectively. Reliability analysis showed that consistency reliability coefficient of the original scale was found to be 0.82.

#### Data Analysis

In this study, SPSS Statistics 27 was used for descriptive statistics and correlation analysis, JASP for reliability analysis, and AMOS Graphics statistical program for structural equation modeling. Firstly, descriptive statistics, reliability analysis, and correlation analysis were conducted. Structural equation modelling was used to investigate whether irrational happiness beliefs and mistake rumination mediate the longitudinal serial relationship between intolerance of uncertainty and family communication. Intolerance of uncertainty was treated as the independent variable, and family communication as the dependent variable.

Parceling technique was used in the analyses of the data. Lee & Whittaker [30] emphasized that this technique should be preferred to reduce measurement errors when single factor scales are used. In addition, Weijters & Baumgartner [44] stated that this method is useful in terms of ensuring measurement normality and reliability. Considering all these reasons, the parceling method was used in this study for the variables of irrational happiness beliefs, mistake rumination and family communication, which consisted of a single factor. Since intolerance of uncertainty has two sub-dimensions, item parceling was not used.

## Results

Table 1 shows descriptive information about the measures, including means, standard deviations, skewness, kurtosis and reliability. Table 1 also shows that there are significant correlations between intolerance of uncertainty, irrational happiness beliefs, mistake rumination and family communication. Intolerance of uncertainty at T1-2 have a significant positive correlation with irrational happiness beliefs and mistake rumination T1-2. At T1-2, however, it has a significant positive correlation with psychological distress. Irrational happiness beliefs T1-2 have a significant positive relationship with mistake rumination T1. Furthermore, family communication T1-2 have a significant negative relationship with intolerance of certainty T2, negative relationship with mistake rumination T2 and positive relationship with irrational happiness beliefs T1.

**Table 1:** Descriptive statistics, reliabilities and correlation findings

Variable	Descriptive Statistics					Correlations							
	Mean	SD	Skewness	Kurtosis	α	1	2	3	4	5	6	7	8
1. Intolerance to Uncertainty T1	40.88	7.71	0.05	−0.54	0.87	–							
2. Intolerance to Uncertainty T2	39.61	8.12	0.03	−0.22	0.90	0.72^**^	–						
3. Irrational Happiness Beliefs T1	9.91	4.51	0.22	−0.66	0.92	0.27^**^	0.09	–					
4. Irrational Happiness Beliefs T2	11.04	4.34	−0.23	−0.81	0.92	0.43^*^	0.41^**^	0.57^**^	–				
5. Mistake Rumination T1	18.85	5.05	0.06	−0.28	0.87	0.55^**^	0.52^**^	0.19^**^	0.30^**^	–			
6. Mistake Rumination T2	17.93	4.77	0.31	−0.06	0.89	0.38^**^	0.54^**^	−0.03	0.09	0.65^**^	–		
7. Family Communication T1	18.60	4.06	−0.42	−0.59	0.90	−0.06	−0.18^**^	0.16^**^	−0.04	−0.18^**^	−0.29^**^	–	
8. Family Communication T2	18.57	3.52	−0.20	−0.56	0.87	0.09	−0.15^**^	0.24^**^	0.15^**^	−0.09	−0.26^**^	0.63^**^	–

### Structural Equation Modeling

First, different measurement models were evaluated. The first wave measurement model was good-fitted to the data (χ^2^ = 60.109, df = 14, χ^2^/df = 4.29, CFI = 0.977, TLI = 0.971, NFI = 0.971, GFI = 0.957, IFI = 0.977 RMSEA = 0.08, SRMR = 0.071) and the second wave (χ^2^ = 34.862, df = 14, χ^2^/df = 2.490, CFI = 0.986, TLI = 0.972, NFI = 0.977, IFI = 0.986, GFI = 0.976, RMSEA = 0.06, SRMR = 0.036). After validating the both measurement model, the longitudinal structural model was investigated.

**Fig. 1:**
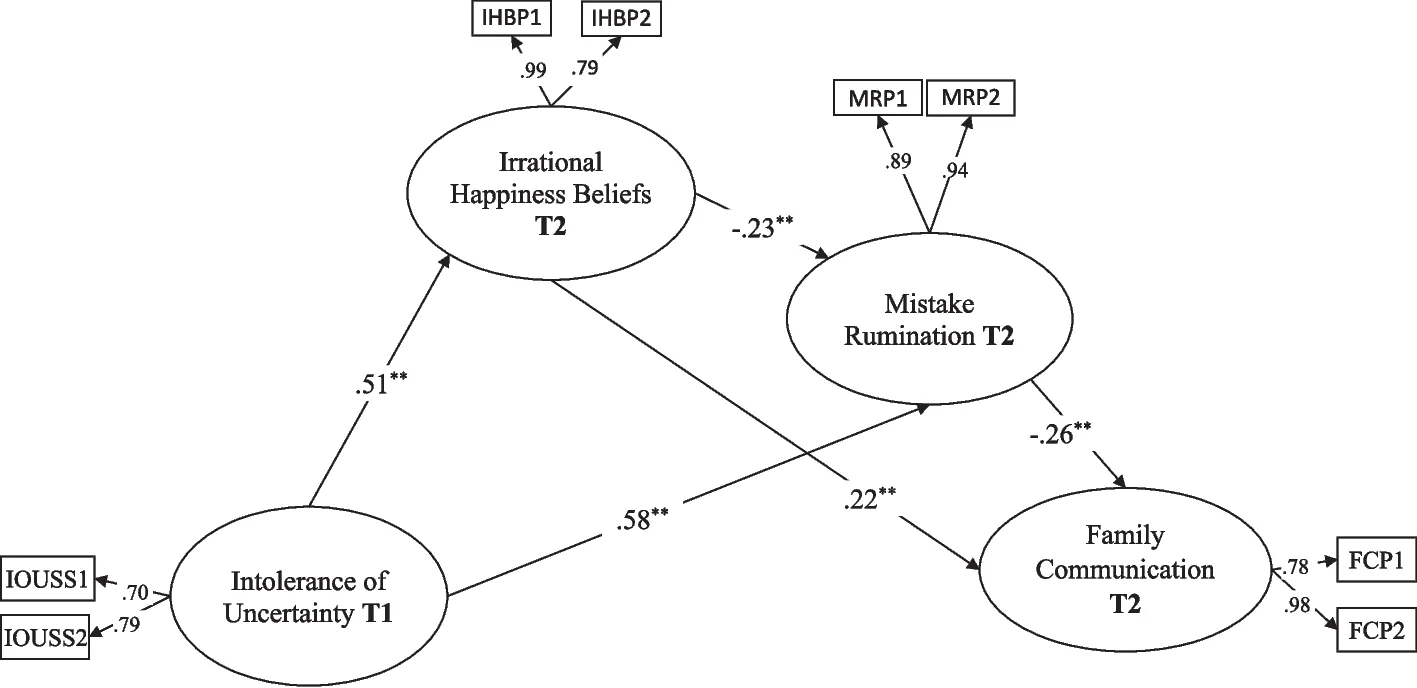
Structural equation modeling for the longitudinal serial mediation model. Note. ^*^ p < 0.05, ^**^ p < 0.01, FCP: parcel of family communication, IHBP: parcel of irrational happiness beliefs, IOUSS: subscale of intolerance of uncertainty, MRP: parcel of mistake rumination

The longitudinal structural model is based on the study hypotheses investigating whether irrational happiness beliefs and mistake rumination has a mediating effect in a longitudinal serial mediation effect on the relationship between intolerance of uncertainty and family communication. The structural model presented a good fit indices to the data. Figure 1 demonstrates that the longitudinal serial mediation model fit was acceptable: χ^2^ = 22.230, df = 15, χ^2^/df = 1.482, CFI = 0.994, TLI = 0.989, NF I = 0.983, IFI = 0.994, GFI = 0.983, RMSEA = 0.04, SRMR = 0.037. Intolerance of uncertainty at T1 significantly predicted irrational happiness beliefs T2, mistake rumination T2; irrational happiness beliefs T2 significantly predicted mistake rumination at T2 and family communication T2; mistake rumination T2 significantly predicted family communication T2.

### Bootstrapping

Bootstrapping technique was applied to the serial longitudinal mediation model, which was hypothetically tested in the study and preferred as the best model in the SEM results. The direct effects was statistically significant. These results reveal the mediating role of irrational happiness beliefs and mistake rumination in the relationship between intolerance of uncertainty and family communication. The results of bootstrapping to provide additional evidence for the significance of direct and indirect paths in the model are presented in Table 2.

**Table 2:** Parameters and 95% C. I. for the paths of the longitudinal final model

Model Pathways	Estimated	95% Confidence Interval	
		Lower	Upper
*Direct link*			
Intolerance of uncertainty T1 → Irrational happiness beliefs T2	0.508	0.386	0.619
Irrational happiness beliefs T2 → Mistake rumination T2	−0.233	−0.413	−0.082
Intolerance of uncertainty T1 → Mistake rumination T2	0.577	0.416	0.753
Mistake rumination T2 → Family communication T2	−0.263	−0.388	−0.099
Irrational happiness beliefs T2 → Family communication T2	0.223	0.119	0.345
*Indirect link*			
Intolerance of uncertainty T1 → Irrational happiness beliefs T2 → Mistake rumination T2	−0.063	−0.140	−0.020
Intolerance of uncertainty T1 → Irrational happiness beliefs T2 → Mistake rumination T2 → Family communication T2	0.012	0.003	0.033
Intolerance of uncertainty T1 → Irrational happiness beliefs T2 → Family communication T2	0.044	0.020	0.082
Intolerance of uncertainty T1 → Mistake rumination T2 → Family communication T2	−0.059	−0.110	−0.020
Irrational happiness beliefs T2 → Mistake rumination T2 → Family communication T2	0.070	0.016	0.165

## Discussion

The family systems approach suggests that the psychological and emotional processes of individuals are shaped by the dynamics of interaction within the family [27]. According to this perspective, family members are mutually interdependent and a change in one individual can affect the functioning of the whole system. This systemic interaction can also directly affect the individual lives of family members. In particular, family harmony is closely related to mutual support and sense of belonging among individuals and is considered as an important indicator of healthy family functioning. In this study, the longitudinal relationships between intolerance of uncertainty, irrational happiness beliefs, mistake rumination and family communication were examined. The study was conducted in a semi-longitudinal design and data were collected over a 5-month period. The findings revealed that irrational happiness beliefs and mistake rumination played a full mediating role in the longitudinal relationship between intolerance of uncertainty and family communication. These results, when evaluated from a family systems perspective, show that individuals'psychological characteristics are related to family interactions over time. The findings of the study are discussed within family systems framework.

To begin with, first hypothesis examined is that Intolerance of uncertainty will longitudinally predict irrational happiness beliefs (H1). According to the findings of the study, the first hypothesis was confirmed. This finding is consistent with the previous findings in the literature [26], [34]. As shown in Fig. 1, individuals who cannot tolerate uncertainty tend to develop rigid and unrealistic beliefs about happiness over time. Being intolerant of uncertainty may cause individuals to overthink their mistakes. Therefore, individuals may ignore the positive and strong aspects of their lives. The protective factor power of family communication in this regard may decrease [31]. Intolerance of uncertainty may affect the hierarchical structure and boundaries within the family. According to the research results, various psychological factors are related to the family process. Over time, different psychological characteristics become associated with family dynamics [50]. Unless individuals are able to tolerate uncertainty, this may affect the stress transference mechanism within the family. This can be understood with the concept of intergenerational transmission in Bowen's family systems therapy. According to Bowen, individuals'ways of coping with stress and uncertainty are learned and transmitted within the family system [10]. Individuals who are anxious in the face of uncertain situations may start to behave more irrational in perceiving happiness and affect family members. According to Minuchin, healthy family systems should have clear but flexible boundaries [33]. If an individual cannot tolerate ambiguity, this may lead to overly rigid or overly diffuse boundaries. Individuals in the family either fuel the conflict by overemotional reactions or cut off communication completely and experience disconnection. Healthy communication within the family contributes to the ability of individuals to express their feelings and thoughts openly and to the development of mutual understanding [41]. However, for some individuals, situations involving uncertainty can be a source of intense anxiety and discomfort. Individuals with low tolerance for uncertainty may create strict rules or develop avoidant communication strategies to increase controllability within the family. Thus, intolerance of uncertainty may become a factor that directly affects communication dynamics and relational harmony within the family [1].

In addition, second hypothesis examined in the study is that Intolerance of uncertainty will longitudinally predict mistake rumination (H2). According to the findings of the study, the second hypothesis was confirmed. This finding is consistent with the previous findings in the literature [12, 29]. Intolerance of uncertainty may negatively affect emotion regulation processes over time and rumination may increase [32]. Individuals who think that happiness should be continuous and perfect may become more fixated on their negative experiences and mistakes [49]. This rumination may cause the person to overthink the mistakes made in the past and to exhibit a rigid and anxious attitude to avoid making mistakes in the future. Constantly dwelling on past mistakes may prevent the individual from establishing flexible and constructive communication within the family. For example, individuals who feel that they cannot make up for past mistakes as a parent or partner may become more withdrawn or avoidant instead of improving their relationships. Individuals may tend to avoid inevitable conflicts and emotional fluctuations within the family, believing that happiness should be continuous [8]. This situation indicates a low level of differentiation. Individuals may become too emotionally dependent on the family system or withdraw too much. Irrational happiness beliefs may trigger oppressive or over-idealizing dynamics within the family. For example, a belief such as “In a good family, everyone should be happy” may prevent the expression of negative emotions. This can lead to the suppression of conflict or the creation of an unhealthy perception of a “perfect family”.

Furthermore, third hypothesis examined in the study is that irrational happiness beliefs and mistake rumination will have a longitudinal serial mediation effect on the relationship between intolerance of uncertainty and family communication (H3). According to the findings of the study, the third hypothesis was confirmed. This finding is consistent with the previous findings in the literature [6, 47]. Being intolerant to uncertain situations may increase rumination [39]. However, this process also affects family dynamics in the future. In the face of uncertain situations, individuals may have rigid happiness beliefs and focus too much on small mistakes. Continuously thinking about past mistakes may have a negative impact on one's communication within the family [17]. Ruminative individuals may blame themselves, be overly critical or avoid communication altogether instead of establishing healthy dialogues within the family. Mistake rumination may disrupt individual boundaries and communication within the family. According to Bowen, an individual's inability to regulate his/her own thoughts and feelings may trigger the triangulation mechanism within the family. For example, because the individual ruminates about past mistakes, he or she may become overly dependent on another family member or avoid communication in order to reduce tension within the family. Mistake rumination may narrow the communication channels within the family and increase the distance between individuals. Mistake rumination may lead to a tendency to “fixate on the past” and “prevent solution-oriented communication” within the family. As a result, the boundaries between family members may break down,individuals may either become overly dependent on each other or experience disconnection [40].

## Limitations and Future Research

There are some limitations despite the significant findings obtained in the study. First of all, data for the study were collected and analyzed at two different time points five months apart. In future research, data could be collected every eight and nine months and repeated in more than two waves (e.g. three or four times) to more clearly establish the causal relationship between the variables more precisely. Another limitation is the research group. Since the data was collected from an adult Turkish sample, its applicability to all cultures and age groups is limited. In future studies, people from different age groups and different cultures can be included. In addition, different demographic information such as different socio-economic levels and education levels can be obtained. Another limitation of the study is that the data were collected using self-report scales. Different data collection methods can be used in future research. Future studies can examine different family dynamics such as family functionality and family belonging. Another limitation is the possibility of social desirability error of the participants, although the data were collected from volunteers. Therefore, different data collection methods should be preferred in future studies to prevent this error.

## Implications

This study examines the relationship between intolerance of uncertainty, irrational happiness beliefs and mistake rumination on healthy family structure. Individuals'intolerance when faced with uncertain situations may lead to the development of irrational happiness beliefs and increased mistake rumination over time. This process may negatively affect communication within the family. Structured intervention programmes may be recommended to increase family members'ability to accept uncertainty and develop resilience. Family members can be directed to establish meaningful and solution-oriented communication instead of overthinking about past mistakes. Within the framework of the family systems approach, brief systemic family therapy [4], structural family therapy [23] and emotion-focused individual therapies [20] have been observed to offer effective results in this context. Moreover, the longitudinal design of the current study allowed us to understand the causal relationships between the variables in more depth. Therefore, the research findings have higher validity than cross-sectional designs in terms of revealing the relationships between variables.

## Conclusion

The study examined the longitudinal relationships between intolerance of uncertainty, irrational happiness beliefs, mistake rumination, and family communication. The results indicated that irrational happiness beliefs and mistake rumination had a significant serial mediating effect in the relationship between intolerance of uncertainty and family communication. Individual characteristics are crucial for maintaining healthy family dynamics. These findings imply that interventions designed to improve uncertainty tolerance and reduce mistake rumination could enhance family communication. Individuals with a higher tolerance for uncertainty may have more flexible happiness beliefs, engage in less mistake rumination, and exhibit healthier communication within the family. Cognitive structures of individuals may influence their roles and boundaries within the family system. Additionally, the family’s communication and conflict resolution dynamics can be shaped by how well an individual copes with uncertainty. Therefore, strengthening skills related to intolerance of uncertainty may be a vital strategy to improve overall family functioning.

## Data Availability

Data will be available on request.

## Declarations

### Pre-registration Statement

This study was not pre-registered.
